# The London Hospital

**Published:** 1893-12-09

**Authors:** 


### HOSPITAL FESTIVALS AND MEETINGS.

### THE LONDON HOSPITAL.

At the General Court of Governors of the London Hospital, held on Wednesday, appropriate reference was made to the late Sir Andrew Clark, and to his services to the hospital, which extended over a period of thirty-nine years.

Mr. J. H. Buxton, who presided, spoke with feeling of the devoted interest which Sir Andrew Clark had always maintained in that hospital. He had not only been senior physician, but the hospital's most active friend. It had been proposed that there should be a permanent memorial of Sir Andrew Clark which should take the form of the endowment of beds or wards in the hospital, which should always be associated with his name. That was the best memorial they could have of such a man. (Applause.) Already a list had been commenced, and the committee would be glad to receive the subscriptions of any governor.

Mr. Hale (Chairman of the Hospital) said he sincerely hoped they would have a whole ward permanently endowed in memory of Sir Andrew Clark. (Applause.)

The Chairman moved the approval of the House Committee's report, which showed that in consequence of the severity of the season the number of patients had been unusually large. The number in the wards at present was 688, which, the chairman said, was really too large. Moreover, the number of deaths during the quarter has been 300, which proved that the cases had not only been numerous but severe. There had also been a good deal of influenza among the nurses, which had made it necessary to draw on the private nursing staff. Mr. Hale seconded its adoption.

Dr. Bedford Fenwick rose to make several suggestions. He thought it would be a great advantage to have the members of the medical staff *ex-officio* members of the House Committee. He strongly recommended that there should be a nursing committee composed of ladies, representatives of the medical staff, on the House Committee. At St. Thomas's, St. George's, and St. Bartholomew's, nursing committees had been found of much service. That hospital was the only one which adhered to the old standard of two years as the period of training for nurses; he thought the term should be lengthened. Dr. Bedford Fenwick then referred to the death of a child in a bath in the hospital, which took place some time ago, as to which particulars have already appeared. He wished that the committee would say what had been done in regard to that matter.

Mr. Yatman wished to know what the average attendance at meetings of the House Committee was; he had been told it was twelve; and that many of the members seldom put in an appearance. There were many things which the House Committee had neglected, such as the reforms recommended by the Lords' Committee, and he suggested that vacancies should be filled up with the names of gentlemen who would be more useful.

The Chairman replied to the criticisms that had been offered. Dr. Fenwick's suggestions had been discussed times without number. As a matter of fact, they were forbidden by their Charter to have members of the medical staff on the House Committee; at the same time, whenever any professional matter arose, the advice of the staff was always sought and obtained. With regard to a Sub-Committee on Nursing, the House Committee really knew best what to keep in their own hands; and they did not consider that this would be a wise move. The system of two years' training of nurses worked well, and produced excellent results; but he had no objection to the matter being—for the tenth or twentieth time—referred to the committee. Mr. Buxton then narrated the circumstances in connection with the death of the child in the bath. It had been clearly brought out at the post-mortem and otherwise that the child had not been drowned, that death had been due to a calculus, and the shock experienced in consequence had been sufficient to bring about the child's death. Mr. Yatman was right as to the average attendance at meetings of the House Committee, which was from twelve to fourteen; and he (the chairman) considered that this number was quite large enough to do the work. The report was then adopted.

The president, office-bearers, and various committees were reappointed for the year. The payment of the usual honorarium of £50 each to members of the out-staff was sanctioned. It was agreed that henceforth in the event of a vacancy in the staff, the House Committee be empowered to consider applications and to make a recommendation to the governors. Candidates will thus have no occasion to engage in the laborious task of canvassing the numerous governors. It was reported that 56 of the sisters and nurses are connected with the Royal National Pension Fund for Nurses, and that the sum of one-half of their premiums, which is paid by the hospital, amounted to £121. The Committee have learned with great satisfaction that Miss Simeon, who received her training as a nurse at that hospital, has established a convalescent home at Ramsgate for patients from the London Hospital. It was stated that the entire cost of the new outbuildings and alterations amounted to £18,568.

